# Psychometric Properties of the Pictorial Scale of Perceived Movement Skill Competence for Young Norwegian Children

**DOI:** 10.1177/00315125241245175

**Published:** 2024-04-16

**Authors:** Håvard Lorås, Ellen Beate Hansen Sandseter, Lise Storli, Rasmus Kleppe, Lisa Barnett, Ole Johan Sando

**Affiliations:** 1 Department of Teacher Education, Faculty of Social and Educational Sciences, 8018 NTNU, Trondheim, Norway; 2 Department of Physical Education and Health, 208420 Queen Maud University College of Early Childhood Education, Trondheim, Norway; 3 Institute for Physical Activity and Nutrition, 2104 Deakin University, Melbourne, Australia

**Keywords:** construct validity, internal consistency, measurement invariance, self-perception, motor skill

## Abstract

The objective of this study was to examine the psychometric properties of the Pictorial Scale of Perceived Movement Skill Competence (PMSC) for young Norwegian children, a scale that is aligned with skills assessed in the Test of Gross Motor Development- Third Edition. We used convenience sampling to recruit 396 Norwegian-speaking children (7–10-year-olds) who completed the PMSC. A confirmatory factor analysis (CFA) confirmed factorial validity for the proposed three-factor model of the PMSC, encompassing measures of self-perceived ball, locomotor, and active play competence. Internal item consistency coefficients of these sub-scales were acceptable, and subsequent measurement invariant analysis found a gender difference such that boys rated their competence higher than girls in running, jumping forward, hitting a ball (racket), kicking, throwing a ball and rope climbing, while girls rated themselves higher, compared to boys, in galloping and skating/blading. Furthermore, there was a slightly better model fit for boys than for girls. Several items were significantly related to children’s age, and the three-factor model exhibited differential age related factor mean differences across older and younger children. Overall, we found the PMSC to have acceptable psychometric properties for confident use in assessing perceived motor competence among 7–10-year-old Norwegian children, though we observed age and gender differences in children’s responses that warrant careful interpretation of results and further research investigation.

## Introduction

The concept of *perceived motor competence,* when applied to children, refers to children’s perceptions of their ability to execute movements (Missiuna, 1998; Raudsepp & Liblik, 2002). This is a multidimensional self-esteem construct that is related to many domains (e.g., social, emotional, cognitive, and academic) but specifically, involves perceived *physical* competence, defined broadly as self-perceptions of observable physical abilities (Harter, 1999). Perceived physical competence might also be considered multidimensional, with perception of motor competence a specific component of overall physical self-perception (Estevan & Barnett, 2018). Perceived motor competence relates to fundamental movement skills purportedly associated with proper future performance of specific motor tasks that will later be needed for formal participation in physical education, sport and/or other physical activities (Barnett, Stodden, et al., 2016). Childhood motor competence may be reflected in the performance of these fundamental movement skills, which have been classified into three categories: locomotion, object control, and stability/balancing skills (Goodway et al., 2021). Perceived motor competence could thus be interpreted as children’s awareness and belief in their own capabilities in these goal-directed fundamental movement skills, including perception of ability to maintain balance (e.g., in landing or walking backward), manipulate an object (e.g., throwing, kicking, or catching) or transport the body from one point to another (i.e., locomotion) (Estevan & Barnett, 2018).

Positive self-perceptions of physical competence have consistently been associated with several positive cognitive, affective, and behavioral outcomes through development (Evans & Roberts, 1987; Robinson, 2011; Sollerhed et al., 2008). In developmental models of interdependent motor skill competence, perceived motor competence has been seen as a mediator between actual motor competence and children’s later engagement and competence in physical activities and sport (Hulteen et al., 2018; Robinson et al., 2015; Stodden et al., 2008). However, a recent systematic review reported indeterminate evidence for this pathway from physical activity to perceived motor skill competence to actual motor skill competence, and there has been no evidence for the reverse pathway (Barnett et al., 2022). Barnett et al. (2022) noted that all studies that found a mediating effect of perceived motor competence had relied on participants who were older children (aged ≥ 9 years). Nevertheless, there is ample evidence to suggest that actual and perceived motor competence both contribute substantively to young children’s health (e.g., Barnett, Ridgers, Zask, & Salmon, 2015; Slykerman et al., 2016; Vedul-Kjelsås et al., 2012). The synergistic nature of the relationship between actual and varied degrees of perceived motor competence suggests that perceived motor competence may promote either positive or negative trajectories of physical activity and later health-related fitness (Kolunsarka et al., 2022; Trecroci et al., 2021). Children with high perceived motor competence have ben found more likely to maintain their participation in physical activities, due to their increased opportunities to develop actual motor competence; reciprocally, increased opportunities to participate in physical activity can help children develop higher perceptions of their motor competence (Robinson et al., 2015; Stodden et al., 2008).

To promote a further understanding of the relationship between actual and perceived motor competence and its significance to health-related developmental trajectories, past researchers have suggested that the assessment of perceived motor competence should depend on a standardized actual motor competence test (Barnett, Lai, et al., 2016; Estevan & Barnett, 2018; McGrane et al., 2016). Following Harter’s recommendation to apply pictorial scales when assessing young children (Harter, 1985, 2012), Barnett, Ridgers, and Salmon (2015) created the *Pictorial Scale of Children’s Perceived Movement Skill Competence* (PMSC). The PMSC was informed by and based on the *Test of Gross Motor Development* (TGMD: Ulrich & Sanford, 1985), a widely used and validated test of actual motor competence in children (Hulteen et al., 2020; Rey et al., 2020; Wiart & Darrah, 2001) that is now in its third edition (TGMD-3; Webster & Ulrich, 2017). In this newest version, six items were added that correspond to children’s active play skills (Barnett, Lai, et al., 2016). Children’s perceptions of their motor competence may vary according to their culture and country of origin, and the PMSC was initially designed and developed for Australian children (Barnett, Ridgers, Zask, & Salmon, 2015).

As of this writing, different versions of the PMSC have been cross-validated for 5-12 year-old children in Portugal (Lopes et al., 2016), China (Diao et al., 2018), Greece (Venetsanou et al., 2018), Spain (Estevan et al., 2018), Brazil (Valentini et al., 2018), Iran (Arman et al., 2021), Italy (Morano et al., 2020) and Canada (Maïano et al., 2022). Similar to the TGMD-3, the initial version of the PMSC was expanded with six additional items intended to assess skills related to playful activities in childhood (e.g., cycling, riding a bike, swimming), and this updated version was examined psychometrically by Barnett, Lai, et al. (2016) who found a superior model fit for a three factor model: active play (6 items), object control-hand skills (4 items) and fundamental movement skills with leg action (8 items). This instrument had relatively high scale score reliability, and validation studies of it suggested general convergence on a two-factor model with locomotor and object control skills (Lopes et al., 2016), that was slightly different from Barnett et al.’s (2016) final solution (object control-hand skills and fundamental movement skills with leg action). However, in separate research, Morano et al. (2020) supported a three-factor model (active play skills, object control and locomotor), and others supported two distinct two-factor models (Estevan et al., 2018; Valentini et al., 2018) involving locomotor (6 items) and object control skills (6 items) and active play skills (6 items) and fundamental movement skills (12 items), respectively. Finally, there have been psychometric analyses of the content validity of the last updated version of the PMSC (Johnson et al., 2016) among samples of Chinese (Diao et al., 2018), Greek (Venetsanou et al., 2018), Spanish (Estevan et al., 2019) and Canadian (Maïano et al., 2022) children, all of which converged on a two-factor solution for the 13 items based on the TGMD-3: ball skills (7 items) and locomotor skills (6 items).

Further examinations of PMSC psychometrics have indicated that subpopulations of children with different characteristics may report varied perceptions of motor competence, which in turn can impact the content validity of a pictorial scale. Gender differences in these children’s perceptions have emerged across validation studies, with boys having reported significantly higher self-perceptions of object control/ball skills than girls (Estevan et al., 2018; Maïano et al., 2022; Morano et al., 2020) and, in Estevan et al. (2019), with boys having presented significantly higher scores than girls not only in ball skills, but also in locomotor skills, and on the global PMSC score. Further examination of potential gender invariance of the PMSC has been somewhat limited, with available data suggesting weak invariance, (i.e., near equivalence of factor loadings across boys and girls (Maïano et al., 2022; Valentini et al., 2018; Venetsanou et al., 2018). These results clearly indicate the importance of testing measurement invariance for the PMSC in the separate populations in which this measure may be used. Strong invariance is an important prerequisite for applying mean subgroup comparisons, while strict invariance is a prerequisite for any form of group comparisons that rely on manifest rather than latent factor scale scores (Meredith, 1993; Millsap, 2012).

There are also indications that children’s age impacts PMSC scores. In Maïano et al. (2022), CFA modelling of data from 5–12-year-old French-speaking Canadian children (*n* = 219) found age related systematic response tendencies and mean latent differences. Specifically, older children tended to score significantly higher on the sliding item and lower on the kicking item, relative to younger children. Furthermore, older children also displayed significantly lower levels of perceived locomotor skill competence relative to younger children). Additional results from Morano et al. (2020) suggested that older children (7 years) tended to present significantly higher scores than younger children (6 years) for object control skills. These findings add to theoretical aspects of self-perception in which such responses might be biased for younger children who have not yet become aware of how they perform, relative to peers (Harter, 2003). There is also evidence that actual motor competence varies as a function of children’s age (Barnett, Lai, et al., 2016).

There are few PMSC validation studies of children in Scandinavia and in southern Europe (e.g., Greece, Portugal, and Spain), even though the PMSC has been used in Finland (Niemistö et al., 2019). In Norway, where the main language is North Germanic (e.g., Norwegian, Swedish, Danish, and Icelandic), there is a need to translate the PMSC with a further non-English validation. Young Norwegian children’s experience of participation in physical education, sport and/or physical activities is also quite different compared to that of children in other countries, and this difference might also influence perceived motor competence when the scale is used among Norwegian children. For example, much of Norwegian culture is connected to formal/informal outdoor activities across all seasons (e.g., swimming, cycling, skiing), and to the major organized children’s sports (e.g., cross-country skiing, soccer, and team handball) (NSC, 2021). Yet, as in many other countries, Norway is still experiencing an obesity epidemic and a decline in physical activity levels (WHO, 2022), highlighting the importance of conducting these PMSC translations.

### Current Study

As stated above, varied cross-cultural research and validation across linguistic groups for various versions of the PMSC have suggested moderate discrepancies in the psychometric properties of this instrument, indicating a need for further psychometric validation of new translations. As no PMSC validation has been conducted for Norwegian children, our main objective in the current study was to examine the psychometric properties of a Norwegian translation of the third version of the PMSC (aligned with the TGMD-3), including the items related to active play skills of 7–10 year-old children.

## Method

### Participants

We recruited a convenience sample (Jager et al., 2017) of 396 Norwegian-speaking children (200 girls, 196 boys) from four elementary schools. We considered this total sample size appropriate for testing the proposed three-factor model of the PMSC (Barnett, Vazou, et al., 2016; Morano et al., 2020) with moderately sized factor loadings (∼.5) and for examining the potential measurement invariance of the factor structure as a function of children’s gender and age (Wolf et al., 2013). The participating children were between 7 and 10 years of age (*M* age = 8.2 years, *SD* = 0.8). The two schools with the most children participating (176 and 115 children, respectively) were from urban environments (two larger Norwegian cities). The two other schools, with 65 and 40 children participating, were smaller and located in rural settings.

### Ethical Considerations

We obtained informed consent from parents/guardians of all participating children before they engaged in any research procedures. We contacted parents via the school’s web-based information system, a provided them information about the project and the possibility of signing an electronic consent form, and, in this way, obtained their written informed consent prior to their child’s involvement in the study. Before initiating any measurements at schools, the children also gave their assent to participate. Our study protocol was approved by the The Norwegian Data Protection Services for Research (ref. no. 784782), and all data and information were managed in accordance with their ethical guidelines. The PMSC data was collected as part of a larger-scale study entitled Virtual Risk Management (ViRMa); for additional specific information regarding the larger-scale study, interested readers are referred to a protocol paper (Sandseter et al., 2023).

### Procedures

#### Translation and Pilot

The translation and adaptation of the PMSC was informed by Gudmundsson´s (2009) guidelines for translating and adapting psychological instruments. One member of our research team (HL) first translated the latest version of the PMSC. Then all five primary researchers conducted an item-by-item discussion of the translated draft. This procedure resulted in a few minor revisions of words and phrases to fit the Norwegian context, and we then recached high agreement amongst the researchers regarding item suitability. Since the scale is based on drawings and not words, we found it unnecessary to conduct a back-translation procedure. In the next step, we included the translated PMSC in pilot work with a sample of *n* = 64 children aged 7–10 from a primary school. All members of the research team participated in assessments. In this pilot, we confirmed that the children in this age group identified the skills and understood the pictures in the PMSC protocol. The data obtained from these children were not included in the sample presented in the current study, though parents of these children had also given their informed consent for the children’s participation. After the pilot work, there were no necessary further adjustments to the Norwegian version.

#### Pictorial Scale of Children’s Perceived Movement Skill Competence (PMSC)

As noted, we applied the latest version of the PMSC that aligns with the TGMD-3 and includes six items involving play skills. We thus assessed children’s perceived motor competence by 13 pictographic tasks, including seven object control skills (catch, bounce, kick, one/two hand strike and overhand/underhand throw), six locomotor skills (run, gallop, hop, jump, slide and skip), and six active play skills (swim, scooter, skate/roller blade, rope climb, bike ride and board paddle). These latter six items were developed to provide a more comprehensive evaluation of children’s perceptions of their motor competence (Barnett, Vazou, et al., 2016), and are therefore an integrated part of the PMSC. For all items, children were first required to choose between two pictures: a cartoon image of a child doing a very good performance of the skill and an image of a child doing a not so good performance of the skill. If children chose the very good performance, they were asked: “are you really good or pretty good at...” If children chose the poorer performance, they were asked: “are you not too good or sort of good at...” This process resulted in a score of 1 (not too good), 2 (sort of good), 3 (pretty good) or 4 (really good) for each skill (Barnett, Lai, et al., 2016).

#### Protocol

All participants’ perceptions of motor competence were assessed individually by the members of the research team in a quiet room at their respective schools. A computer tablet (iPad Pro 12.9″) was applied to present the figures and record the children’s responses using SurveyXact software (Rambøll Management Consulting, Oslo, Norway), and the instructions and items of the PMSC were read aloud by the interviewer. First, the children were asked whether they had or had not already tried the skill depicted in the cartoon drawings. If they had never tried the specific item, they were asked to imagine themselves doing it. If difficulties emerged in understanding or recognizing a skill amongst those who had never tried it, a physical demonstration of the illustration in the figures was proposed. This last scenario took place very rarely, as most of the items were recognized by the full sample of children. Finally, for each item, children were asked to point out their response, which was then recorded on the tablet by the interviewer. The total administration of the PMSC rarely exceeded 8–10 minutes to complete.

### Statistical Analyses

We conducted descriptive analysis to report percentages of boys and girls according to “how good” they thought they were at each of the 19 skills (i.e., ‘really good,’ ‘pretty good,’ etc.). To investigate the relationship between self-ratings for each item and the child’s age and gender, we performed a regression analysis (Mehmetoglu & Jakobsen, 2017), with each item regressed on the child’s age and gender.

The initial a priori theoretical model consisted of 19 items divided into three hypothesised factors (6-7-6 items): *locomotion* (run, gallop, hop, leap, jump and step slide), *ball skills* (hitting a ball with one (racket) or two hands (baseball bat), bouncing, catch, kick, underhand and overhand throw) and *active play* (cycle, skate, roll, board paddle, swim, and climb). To assess the internal consistency or composite reliability of the measurement, we calculated Cronbach’s Alpha for each subset of items: seven ball skill items, six locomotor skill items, and six active play skill items. This analysis aimed to determine how well the items within each subtest measured their respective skills.

We applied confirmatory factor analyses (CFA) (Brown, 2014) to evaluate the proposed model. All CFA analyses used robust maximum likelihood estimation with robust standard errors to account for the ordinal level items. The evaluation of the model utilized several indices, including the Root Mean Square Error of Approximation (RMSEA), Standardized Root Mean Square Residuals (SRMR), Comparative Fit Index (CFI) and Tucker-Lewis Index (TLI). Acceptable model fit was determined based on specific criteria: RMSEA <0.1, SRMR <0.1, CFI >0.9, and TLI >0.9 (Mehmetoglu & Jakobsen, 2017). To examine the factor loadings, *R*^2^ estimates (item >0.25) and standardised factor loadings (item >0.40) (Brown, 2014) were examined. If the proposed theoretical model did not meet the specified acceptable model fit indices, alternative models were explored using exploratory factor analysis (EFA) (Brown, 2014).

We evaluated the influence of gender and age on the factor structures within the models. To achieve this, we employed multiple-group CFA invariance evaluation (Brown, 2014) to determine whether the factor structure remained consistent across different groups (Van De Schoot et al., 2015). This analysis allowed us to assess whether the relationships between the observed indicators and the latent factors are comparable and invariant across various groups. In this study, the following analyses of measurement invariance was performed: (a) equal form, (b) equal factor loadings, (c) equal intercepts, (d) equal indicator residual variances and (e) equal factor means (Brown, 2014). We used STATA MP version 18 (STATACorp) software for all statistical analyses.

## Results

The summed scores of the 19 items indicated that these 396 children, on average, rated their perceived motor competence as mostly good (*M* rating = 2.9, *SD* = 0.4), with a range between 1.7 and 4. The participating children rated their perceived skills to be highest for the items measuring running (*M* = 3.5, *SD* = 0.7) and bike riding (*M* = 3.5, *SD* = 0.7), and lowest for the item of hitting a ball with two hands (baseball bat) (*M* = 2.1, *SD* = 1.0). Descriptive statistics and the percentage ratings of boys and girls for “How good” the children thought they were at each skill are presented in Table 1.

**Table 1.** Means, Standard Deviations (SD) and Percentages of Boys and Girls on Self-Ratings of “How Good” They Were at Each Skill (*n* = 396).

Subscales and Items	Mean	SD	Not too good (%)		A little good (%)		Good (%)		Very good (%)	
			Boys	Girls	Boys	Girls	Boys	Girls	Boys	Girls
Locomotor skills										
Gallop	2.4	0.9	20	12	48	40	22	32	10	16
Skip	2.8	0.9	11	8	23	22	37	46	29	24
Step and slide	2.9	0.9	7	4	30	24	35	46	28	26
Jumping forward	3.0	0.8	4	6	16	23	44	51	36	20
Hop	3.2	0.7	1	2	12	14	50	47	37	37
Run	3.5	0.6	0		5	7	36	45	59	47
Ball skills										
Hitting a ball two hands	2.1	1.0	26	44	34	34	25	16	15	6
Hitting a ball one hand	2.4	1.0	15	23	32	41	35	25	18	11
Underhand throw	2.7	0.9	10	13	24	36	40	39	26	12
Catch	3.0	0.9	4	6	21	22	37	40	38	32
Kick	3.2	0.9	3	6	8	26	30	35	59	33
Overhand throw	3.2	0.9	3	9	9	14	39	39	49	38
Bouncing a ball	3.3	0.8	3	3	11	15	40	38	46	44
Active play skills										
Skate and/or blade	2.2	1.1	46	17	27	35	15	25	12	23
Rope climb	2.3	1.0	23	27	35	42	24	17	18	14
Board paddle	2.8	1.0	13	10	21	25	39	36	27	29
Swim	3.0	0.9	11	8	15	15	37	43	37	34
Scooter	3.2	0.8	5	2	19	15	35	40	41	43
Bike ride	3.5	0.7	4	3	7	5	27	29	62	63

Boys scored significantly higher for the locomotor items or running (β = 0.18, *p* = .005) and jumping forward (β = 0.29, *p* = .000) than girls, while girls scored significantly higher than boys for the locomotor item gallop (β = −0.30, *p* = .001). The items hop, skip, step and slide were not significantly different statistically across gender groups. For most items related to ball skills, boys scored significantly higher: (a) for hitting a ball with two hands (baseball bat) (β = 0.42, *p* = .000); (b) for hitting a ball with one hand (racket) (β = 0.33*, p* = .001); (c) for kicking a ball (β = 0.52, *p* = .000),; (d) for underarm throw (β = 0.32, *p* = .001); and (e) for overhand throw (β = 0.29, *p* = .001). Bouncing and catching a ball were not significantly different across gender. The active play skills of bike riding, swimming, scootering, and board paddling were not statistically different across gender, but boys rated their active play skills significantly higher than did girls for the rope climbing item (β = 0.20, *p* = .049), and girls scored significantly higher than boys for their perceived skating and blading competence (β = −0.62, *p* < .001).

Age was negatively related to the locomotor skills of jumping forward (β = −0.15, *p* = .001), and stepping and sliding (β = −0.16, *p* = .002). The locomotor skills of running, galloping, and hopping were not significantly related to age. Within ball skills, only kicking a ball was negatively associated with age (β = −0.17, *p* < .001). Age was not significantly related to hitting a ball with two hands (baseball bat), hitting a ball with one hand (racket), bouncing a ball, catching, underhand throwing, or overhand throw. The active play skills of bike riding, swimming, scootering, skating, and blading were also statistically unrelated to the child’s age, but age was negatively associated with the child’s self-perceived active play skill of rope climbing (β = −0.13, *p* = .019) and was positively associated with board paddle (β = 0.12, *p* = .035).

The Cronbach alpha coefficients obtained for locomotor (α = 0.56), ball (α = 0.67) and active play skills (α = 0.59) indicated a moderate degree of internal consistency. Inter-item correlations on subscales were also computed. The average inter-item correlation was lower than 0.20 for locomotor skills (0.17) and active play skills (0.19), while ball skills (0.22) were within the ideal range of average inter-item correlation of between 0.20 and 0.40. Pairwise correlational coefficients for items within the locomotor were mostly between 0.10 and 0.20, with the highest correlation between jumping and running (r = 0.27, *p* < .001) and the lowest between running and galloping (r = 0.06, *p* = .256). For the items measuring ball skills, the pairwise correlation coefficients were somewhat higher, ranging from the highest of hitting a ball with one hand (racket) and hitting a ball with two hands (baseball bat) (r = 0.32, *p* < .001), to the lowest of bouncing a ball and underhand throw (r = 0.11, *p* < .032). Among the active play items, a moderate correlation was found between the items swimming and board paddling (r = 0.42, *p* < .001), while the other correlations were below 0.23, with the weakest between swimming and skating (r = 0.10, *p* = .057).

Next, we evaluated the proposed three-factor model (locomotor, active play, and ball skills) of the PMSC using confirmatory factor analysis (CFA). The exogenous latent variables could be correlated, and locomotor skills were closely associated with both ball skills (β = 0.72, *p* < .001) and active play (β = 0.76, *p* < .001). Children’s perceived ball and active play skills were also significantly associated (β = 0.58, *p* < .001). The model fit measures for the proposed three-factor model suggest that the model could be improved. The chi-square value of 281 with 149 degrees of freedom indicated that the observed data deviated significantly from the expected values, based on the specified model. The RMSEA (0.0047) and SRMR (0.054) suggested a reasonably good model fit. However, the CFI (0.85) and TLI (0.82) fell slightly below the commonly accepted threshold of 0.90. Furthermore, several standardized factor loadings were below the threshold of 0.40 (Table 2): two items of the locomotor skill scale (run and gallop), one of the ball skills (kick), and two of the active play skills (skating/blading, bike riding). On examining the *R*^2^ estimates (Table 2), none of the items of the locomotor and active play skill subscales exceeded the set threshold of 0.25. Only three out of seven items of the ball skills scale exceeded the set threshold. These results indicated a moderate model fit and internal consistency for the suggested three-factor model.

**Table 2.** Standardized Factor Loadings (λ) and *R*^2^ Estimates From the Three-Factor Solution of the Pictorial Scale for Perceived Movement Skill Competence Using Confirmatory Factor Analysis (*n* = 396).

Items	Locomotor skills		Ball skills		Active play skills	
	*λ*	*R*²	*λ*	*R*²	*λ*	*R*²
Run	0.36	0.13				
Gallop	0.38	0.14				
Hop	0.44	0.19				
Skip	0.43	0.19				
Jumping forward	0.47	0.21				
Step and slide	0.43	0.19				
Hitting a ball two hands			0.50	0.25		
Hitting a ball one hand			0.50	0.25		
Bouncing a ball			0.45	0.20		
Catch			0.50	0.25		
Kick			0.37	0.14		
Underhand throw			0.48	0.23		
Overhand throw			0.49	0.24		
Bike ride					0.39	0.15
Swim					0.45	0.20
Rope Climb					0.49	0.24
Scooter					0.44	0.19
Skate and blade					0.39	0.15
Board paddle					0.48	0.23

Following the observed moderate model fit, we conducted an exploratory factor analysis (EFA) to further investigate the dimensionality and explore alternative measurement models for all items. A principal factor analysis revealed the presence of a single factor with an eigenvalue above 1.0, indicating its significance in explaining the variance within the scale. Further examination of a scree plot, extracted variance, and parallel analysis supported the significance of this factor. The identified factor exhibited an eigenvalue of 3.0, explaining 31% of the variance in the scale. However, all items with rotated factor loadings above 0.3 for this factor were only associated with the proposed ball skills scale: bouncing (0.51), catching (0.46), overhand throw (0.42) and kicking (0.40). The internal consistency reliability, as measured by Cronbach’s alpha coefficients, for these four items was found to be 0.57. Considering that the alternative model solely consisted of the four ball skills items, this finding indicates that EFA analysis of all items did not reveal the presence of an alternative measurement model that comprehensively captured various aspects of children’s perceived motor competence beyond the proposed three-factor model. Consequently, further exploration of this alternative model was deemed unnecessary.

In further analysis, we conducted a multi-group CFA using the proposed three-factor theoretical model to examine measurement invariance for the factor structure. Initially, we fitted separate models for boys and girls, as presented in Table 3. The three-factor model showed a CFI of 0.85, TLI of 0.82, RMSEA of 0.047 and SRMR of 0.054. When analyzing boys separately, the three-factor model demonstrated a better fit with higher CFI (0.91) and TLI (0.89), and lower RMSEA (0.036), compared to the overall model. These results indicate that the three-factor model fits well for boys. In contrast, the three-factor model exhibited a poorer fit for girls, as evidenced by lower CFI (0.80) and TLI (0.78), and higher RMSEA (0.055) and SRMR (0.066), compared to the overall model. This suggests that the three-factor model is less suitable for capturing the underlying factor structure among girls. These findings indicate an unequal form or differential factor structure between boys and girls. Notably, the factor loadings were equal for boys and girls (χ2diff = 18.2 (16), *p* = .31). However, further analysis revealed significant differences in other measurement aspects between boys and girls. Specifically, the intercepts showed significant variation (χ2diff = 104 (16), *p* < .01). Moreover, there were substantial differences in residual indicator variances (χ2diff = 41.1 (19), *p* < .01) and factor means (χ2diff = 49.1 (3), *p* < .01) between boys and girls. Furthermore, boys scored significantly higher for latent variable ball skills (β = 0.34, *p* < .001), but not for active play and locomotor skills. These differences indicated that the measurement properties of the three-factor model differ significantly between the two groups.

**Table 3.** Goodness-Of-Fit Indexes of the Three-Factor Model for the Pictorial Scale for Perceived Movement Skill Competence for Full and Sub-samples Using Confirmatory Factor Analysis.

Model	N	χ2 (df)	CFI	TLI	RMSEA	SRMR
Three-factor model	396	281 (149)	0.85	0.82	0.047	0.054
Three-factor model boys	196	186 (149)	0.91	0.89	0.036	0.058
Three-factor model girls	200	240 (149)	0.80	0.78	0.055	0.066
Three-factor model youngest	195	208 (149)	0.85	0.82	0.045	0.064
Three-factor model oldest	201	215 (149)	0.86	0.84	0.047	0.066

*Note.* χ2 = chi-square; df = degrees of freedom; CFI = comparative fit index; RMSEA = Root Mean Square Error of Approximation; SRMR = standardized root mean residual; TLI = Tucker-Lewis index.

Probability level *p* < .05.

To investigate the impact of age on model invariance, we examined the three-factor model separately for the youngest (age <8.75) and oldest (age >8.75) children, as presented in Table 3. Within the youngest children subset, the three-factor model demonstrated similar goodness of fit with comparable values for CFI (0.85), TLI (0.82), RMSEA (0.045), and SRMR (0.064) when contrasted with the overall model. Correspondingly, the three-factor model exhibited a similar level of fit within the oldest children subgroup, where CFI (0.86), TLI (0.84), RMSEA (0.047), and SRMR (0.066) approximated the values from the overall model. These outcomes imply a consistent model fit across the two distinct age clusters included in this study. Further assessments of invariance demonstrated uniform factor loadings across the age groups (χ2diff = 14.3 (16), *p* = .58). However, the intercepts test showed a significant difference between the age groups (χ2diff = 34.8 (16), *p* < .01). Similarly, the equal error variances test yielded a significant difference between the ages (χ2diff = 42.6 (19), *p* < .01). Lastly, the factor means were equal for the two age groups (χ2diff = 7.8 (3), *p* = .05). These findings suggest that the three-factor model exhibited differential measurement invariance across the two age groups.

## Discussion

Our principal aim in the current study was to verify psychometric aspects of a Norwegian adaptation of the PMSC (aligned to the TGMD-3), including the items for children’s active play skills, among a sample (*n* = 396) of 7–10-year-old Norwegian children from primary schools. Our results indicated that these children rated their perceived motor competence primarily as good or very good. Nonetheless, we observed a higher degree of inter-item variance in responses for seven items (galloping, skipping, hitting a ball, underhand throwing, rope climbing, skating/blading and board paddling) (see Table 1). As depicted in Tables 2 and 3, our confirmatory factor analysis (CFA) indicated moderate support and goodness-of-fit for the proposed three-factor model for the Norwegian version of the PMSC encompassing measures of perceived ball, locomotor, and active play skills competence. The internal consistency reliability coefficients of these sub-scales were in the range of 0.56–0.67 (Cronbach’s alpha), reflecting relatively moderate-to-low inter-item correlation coefficients within subscales. However, there were statistical indices of measurement invariance in the study sample, as boys rated their competence higher compared to girls for five items (running, jumping forward, hitting a ball, kicking, throwing and rope climbing). Girls, on the other hand, rated themselves higher compared to boys for galloping and skating/blading. The CFA indicated a slightly better model fit for the boys. For five of the items, the children’s responses were significantly related to their age, and the three-factor model exhibited differential measurement invariance and factor mean differences across older and younger children.

The inter-item frequency distributions of children’s self-perceptions in the current study, depicted in Table 1, indicated that around two thirds of the children rated themselves as good/very good across items. These patterns of results were aligned with previous validation studies of the PMSC, both for earlier versions of the scale aligned with the TGMD-2 (Barnett, Vazou, et al., 2016; Estevan et al., 2018; Valentini et al., 2018) and the latest version applied in the current study that was aligned with the TGMD-3 (Arman et al., 2021; Diao et al., 2018; Venetsanou et al., 2018). In the current sample of 7–10 year-old Norwegian children, however, two locomotor items (galloping and skipping), three ball skills (one and two hand hitting a ball, underhand throwing), and three active play skills (rope climbing, skating/blading and board paddling) had higher inter-item variance. Differences in perceptions of some of these items can possibly be explained by the culture in Norway associated with physical activities: for the one-hand hitting item, as the picture depicts a child using a racket, and for the two-hand hitting, the drawing shows a child swinging a baseball bat. These motor skills are rarely practiced in organized sport among Norwegian children, which is also the case for skating/blading and board paddling skills (NIH, 2021). It should be noted, however, that 40–50% of Norwegian children have been reported to do some recreational and non-organized ice skating during the winter season, which might evoke reflections of skill pertaining to the skating/blading item (Vaage, 2015). Furthermore, although rope climbing can be a skill practised in physical education lessons in school, it is not a mandatory activity, and many Norwegian children might not have gained any experience with this item. These results thus mirror findings that children show greater variation in reporting for those PMSC items with which they have less experience (Barnett, Ridgers, Zask, & Salmon, 2015; Barnett, Vazou, et al., 2016; Lopes et al., 2016). The locomotor items of galloping and skipping have been discussed in other independent validation studies, and there are indications that these items depict skills that might be less well-known or practised, as well as being harder to distinguish from each other, and are consequently not as well-recognized by children as the other skills (Barnett, Ridgers, Zask, & Salmon, 2015; Diao et al., 2018; Estevan et al., 2018; Lopes et al., 2016). In a recent French validation study, the galloping item was also found to be suboptimal in their CFA model (Maïano et al., 2022). This seems to be a cross-cultural finding which warrants further examination.

The CFA reported in the current study for the model with locomotion, ball skills and active play as the three theoretical factors (6-7-6 items) indicated moderate support for the measurement model. The standardized factor loadings depicted in Table 2 (range λ = 0.3–0.5) are lower than what was found in Iranian children (range λ = 0.6–0.7, Arman et al., 2021), although similar in range compared to samples of Greek (range λ = 0.3–0.6, Venetsanou et al., 2018) and Canadian children (range λ = 0.3–0.6, Maïano et al., 2022) for the latest version of PMSC aligned with the TGMD-3. Furthermore, the reported standardized factor loadings for the previous version of PMSC based on the TGMD-2 is in the range of λ = 0.4–0.7 (Barnett, Lai, et al., 2016; Estevan et al., 2018; Lopes et al., 2016; Valentini et al., 2018). Other support for the proposed three-factor model was found in goodness of fit indices, in which RMSEA/SRMR was within proposed cut-off values and the CFI/TLI was slightly below some recommended cut-offs (see Table 3). These results from our sample of Norwegian children also reflect other modelling work on the current (Arman et al., 2021; Diao et al., 2018; Maïano et al., 2022; Venetsanou et al., 2018) and previous versions of the PMSC (e.g. Lopes et al., 2016; Valentini et al., 2018), in which the predominant aspect of various SEM approaches and their respective reported model fit indexes suggested support for the locomotion, ball and active play skill factors.

Our statistical modelling results (see Table 2), and those of others (Diao et al., 2018; Estevan et al., 2019; Maïano et al., 2022; Morano et al., 2020; Valentini et al., 2018; Venetsanou et al., 2018) suggest relatively consistent evidence for the theoretical model of PMSC (including active play skills, ball skills and locomotor skills) across cultural and linguistic adaptations. These three components thus seem to be developmentally applicable constructs that capture representative aspects of perceived motor competence in relatively diverse samples of young children. The consistent modelling results across populations also indicates that these perceptions of ability in proposed fundamental movement skills are basic and specific skills (Estevan & Barnett, 2018; Goodway et al., 2021) that children easily recognize and potentially apply in their play and daily activities before potentially learning more specific skills through formal participation in physical education, sport and/or physical activities.

The internal consistency reliability coefficients obtained in the current study across factors (α = 0.6 – 0.7) were somewhat lower compared to estimates found in previous cross-linguistic investigations of the recent PMSC version, which have been in the range of α = 0.6–0.8 (Arman et al., 2021; Diao et al., 2018; Maïano et al., 2022; Venetsanou et al., 2018). The coefficients obtained in our study, and in some of the results of other studies, thus suggest estimates below the commonly reported threshold of 0.7 (Bland & Altman, 1997), which might indicate questionable internal consistency. However, the interpretation of such statistical indexes is highly debated (Cortina, 1993; Sijtsma, 2009), and there are no standard values for acceptable internal consistency that can be applied in every context (Bland & Altman, 1986). Perceived motor competence captured by the PMSC is based on self-evaluation of individual performance levels in the TGMD-3 and active play tasks, and it is well-known that assessment of actual motor performance is particularly prone to substantial inter- and intra-individual variability that, among other things, can be displayed as low correlations between performance for different motor tasks (Lorås & Sigmundsson, 2012; Sigmundsson et al., 2021). Furthermore, within-trial individual variability can be more substantial for motor tasks compared with cognitive tasks (Lövdén et al., 2008), which suggests that internal consistency statistics both within and between performance domains are not necessarily comparable. In view of the nature of the motor tasks by which children self-evaluate their competence level in PMSC, one cannot necessarily expect young children to provide a consistent rating of competence level across all items within a specific factor. Swimming and rope climbing, for example, are very different and specialised motor skills for which competence level is highly dependent on the children’s previous experience in physical education or sport contexts, for example. Against this background, some degree of statistical inter-item variation in children’s ratings might be expected for scales such as the PMSC.

Our results are generally consistent with those from previous validation efforts, in that boys typically perceive themselves as more skilled in ball handling competence than girls (e.g., Barnett, Lai, et al., 2016; Estevan et al., 2018; Maïano et al., 2022). In the current sample of Norwegian children, boys also reported greater competence in two locomotor items (running and jumping forward). These differences in perceptions of skill align with differences in measures of actual motor competence based on the TGMD-2: in a meta-analysis of scores from 25 different countries across six continents, weighted mean scores indicated that the boys in each age range (3–5, 6–8, 9–10 years and overall) exhibited higher levels of greater object control skill proficiency compared to their female counterparts (Bolger et al., 2021). A novel finding of the current study was that girls rated themselves higher compared to boys for galloping and skating/blading. These latter differences are not reliably explained by any cultural factors, although there are some indications that ice skating as a recreational activity is more popular among girls in Norway (Vaage, 2015). The results might therefore reflect that in the current sample of Norwegian children the measurement properties of the three-factor model differed significantly as a function of gender: CFA suggested a better fit for the three-factor model in boys (see Table 3). This finding aligns with results obtained by Maïano et al. (2022) from their sample of Canadian children, although Venetsanou et al. (2018) did not find any evidence of measurement invariance of the PMSC factors as a function of gender in young Greek children. Our slightly better fit for the three-factor model in boys (Table 3), also reported by Maïano et al. (2022), does not indicate that the PMSC cannot be used confidently for both boys and girls since the overall goodness-of-fit indexes were within the boundaries of acceptable criteria for model fit. Rather, these results indicate that decisions on comparing boys and girls using latent PMSC variables must be based on sample-specific cultural and linguistic validation efforts, relating to whether any measurement invariance needs to be accounted for in statistical procedures.

We found that older children reported significantly lower levels of perceived motor competence relative to younger children for five different items, and that the CFA indicated differential measurement invariance and factor mean differences across age (Table 3). Older children were thus more consistent in their perceptions across PMSC items. These findings align with statistical modelling efforts reported by Maïano et al. (2022), who reported similar results as a function of age. Such findings might be explained by theoretical perspectives on children’s self-perceptions: younger children might display inflated and less consistent responses as they have less experience with, and awareness of, peer-to-peer comparisons and are less inclined to reflect upon their own motor competence levels (Harter, 2003). However, in a meta-analysis of 69 studies of the association between actual motor competence and perceived motor competence/physical self-perception, significant pooled effects were found for locomotor, object control, stability/balance, and sport-specific competence, in which age (3–24 years old) did not appear as a significant moderator for any of the domains (De Meester et al., 2020). Thus, the authors of the meta-analysis did not find support for less consistent perceptions in younger children, although the analysis was somewhat limited in terms of sample size for older children. In either case, as with the gender differences in PMSC responses discussed above, it is necessary to account for children’s age when interpreting perceived motor competence scores.

### Limitations and Directions for Further Research

Our study had methodological limitations that warrant further commentary. Of importance, we presented no evidence of the test-retest reliability or aspects of concurrent/divergent/predictive validity of the Norwegian version of the PMSC. These psychometric properties are currently being investigated among Norwegian children with combinations of other self-report measures and objective measures of gross motor competence. In further work with perceived motor competence and instruments such as the PMSC, researchers should consider that boys and girls at the age of 7–10-years might be different in how consistently they rate their own perceived motor competence item-by-item, and that boys seem to consistently rate themselves as more competent compared to girls (see table 1). Such baseline differences are especially important to consider and include in analytical approaches when changes in perceived motor competence are investigated in interventional and/or developmental work with this age group of children.

## Conclusion

In conclusion, these results reveal that internal psychometric properties (factor validity, internal consistency reliability and measurement invariance) of the Norwegian translation of the PMSC are sufficient for further work with this measure of perceived motor competence and some of its underlying constructs (locomotion, ball skills, active play) in Norwegian 7-10 year-old children. Furthermore, these data align with previous cultural/linguistic validations of the PMSC in that age and gender potentially impact measurement invariance and factorial differences when children rate their perceived motor competence, and these differences need to be considered in future studies of this construct (Barnett et al., 2016).

## Data Availability

The data that support the findings of this study are available from the corresponding author, [HL], upon reasonable request.
